# Medical Opinion and Movement

**Published:** 1906-09-29

**Authors:** 


### Medical Opinion and Movement.

There is no doubt that the prevailing opinion among general practitioners throughout the country is that cancer is greatly upon the increase. The majority of the cases occur among women; but men are being attacked in increasing numbers every year. In one family recently the medical attendants noticed that the wife died of cancer, and, when the widower subsequently married two years afterwards, the second wife, though a strong and healthy woman, died also from cancer. Within a further eighteen months the husband, who continued to reside in the same house, died of cancer too. It is time that a commission was appointed to inquire into and report whether cancer is contagious or not.

The "silly season" has produced one relatively new gooseberry in the shape of a cry that in the near future England will lack sufficient doctors to supply the public needs. The only justification for this assertion would seem to lie in the fact that hospital abuse continues to increase at such a pace that already in the Metropolis the new hospital patients treated each year exceed by many thousands the whole number of persons by which the total population of the community has grown in the same period of twelve months. This cry of a future dearth of doctors is the only justification for the existing hospital abuse which we have come across for a long time, but it does not commend itself to our judgment as sound or reasonable.

There is however another and even more serious aspect of this question. The average practitioner is so harassed by the struggle for existence which he often has to face, that there is a danger that he is losing his nerve, and may consequently sacrifice the confidence of his patients, and so lower the profession as to permanently diminish, if he does not even destroy, the authority of the physician. Patients complain frequently that the practitioner they have consulted gave them the impression that he was striving all the time to ascertain what advice they wished him to give them, rather than to make his diagnosis and issue his instructions as to treatment. It will be a bad day for medicine and the medical profession when patients generally come to the conclusion that medical opinion is not mandatory but suggestive. Christian Scientists have indirectly raised this question in the United States by causing insurance companies to consider whether policy-holders who refuse the services of a practitioner in illness offer a safe risk for insurance companies. The conclusion so far arrived at appears to be that when a Christian Scientist is really ill, whatever opinion he may hold in health, he does, in fact, seek skilled medical aid. Be this as it may, we are confident that it is bad business in every sense for a practitioner not to give a clear diagnosis of every case and to make his treatment mandatory.

There can be no doubt that bird life is one of the most attractive features of the country districts throughout Great Britain. The Rev. J. G. Cornish has shown many indications that the birds of Europe regard the British Isles as a country which is steadily improving both as a residential district and a place to visit. Twenty years ago no one would have dreamed of what may be seen by the observant in London to-day in regard to bird life. We have sea-gulls taking food from the hands of men on London Bridge, and carrion crows establishing themselves in Regent's Park and finding plenty of nests to rob there. In the parks, and even in some of the very few gardens which remain in central London, pigeons and doves habitually build and rear their young. The increase in the number of cuckoos is a marked feature of the effects of the Small Birds Protection Acts, as may be realised by the observant who are fond of natural history and will take the trouble to wander along the hedgerows during the breeding season in country districts. All these signs of confidence and development manifested by birds of all kinds must prove welcome to the thoughtful amongst us, who love their country and take every opportunity of studying and enjoying its beauties.

The time has certainly arrived when the Government should set its hand to consolidate the health legislation in this country, especially in regard to the powers conferred upon urban and rural authorities. In the absence of such consolidation each county council and authority seeks legislation from time to time to effect small but important improvements in sanitary matters throughout their area. This is a costly, cumbersome way of attempting to meet the needs of the population in regard to hygiene. It has the disadvantage that progress is not universal, and that it is confined mainly to the more actively administered authorities, who take an intelligent interest in the work which Parliament has entrusted to them. Legislation which confers powers upon authorities to prohibit the supply within the area of milk from a dairy situated within or outside its limits when the medical officers are of opinion that infectious disease has been caused or is likely to be caused by the consumption of such milk; power to require dairymen to supply lists of their customers and the sources from which their milk supply is obtained, and to grant compensation in certain cases; power to enable authorities to set up milk depots for the supply of milk suitable for infants; power to require owners to make reasonable provision for the supply of water to each floor in tenement houses, and to provide in such houses for the storage and cooking of food, are a few only of the provisions which a consolidated and amended Health Act ought to contain. The advance which has been made in hygienic knowledge in recent years, and the important bearing it has upon the health of communities, especially in large cities, should cause the Government to take action in this matter at the earliest possible moment.

